# Experimental study on compression mechanical characteristics of filled rock joints after multiple pre-impacts

**DOI:** 10.1038/s41598-022-15849-5

**Published:** 2022-08-10

**Authors:** Shaobo Chai, Yongsheng Jia, Yuxiang Du, Bo Hu, Xianpeng Li

**Affiliations:** 1 State Key Laboratory of Precision Blasting of Jianghan University, Wuhan, 430056 China; 2 Huebi Key Laboratory of Blasting Engineering of Jianghan University, Wuhan, 430056 China; 3 School of Civil Engineering, Chang’an University, Xi’an, 710064 China

**Keywords:** Solid Earth sciences, Engineering

## Abstract

The prefabricated artificial filled jointed rock specimens are impacted by a self-made drop hammer impact device for many times, and the specimens with different degrees of cumulative damage characteristics are obtained. Then, the static and dynamic compression mechanical properties are studied by using universal testing machine and SHPB device. Through the static compression test, the strength and deformation characteristics of jointed rock specimens after multiple impacts are obtained, and the influence of the damage degree of jointed rock specimens characterized by wave velocity on the compressive strength of filled joints is analysed. Based on the results of SHPB impact test, the dynamic strength and deformation evolution, wave propagation law and energy dissipation law of filled joints after multiple impacts are analysed. During the SHPB test, the impact failure process of rock specimens is recorded by a high-speed camera. The experimental results show that the damage degree of jointed rock samples increases nonlinearly after multiple impacts. The attenuation laws of static strength and dynamic strength of rock samples under the same damage evolution conditions are different. With the increase of impact times, the failure mode of jointed rock samples after damage is simpler and tends to compression failure.

## Introduction

Filled joint is an important medium in engineering rock mass. Its failure mode, strength and deformation characteristics under external load will directly affect the design and construction of engineering. In the process of nuclear power foundation excavation, deep tunnel excavation, construction of high-level radioactive waste geological disposal repository and water conservancy and hydropower project construction, the stress wave generated by blasting will inevitably act on the filled jointed rock mass. The lack of accurate understanding of the dynamic characteristics of the filled joint has led to many engineering accidents. Therefore, the dynamic characteristics of filled joints and the propagation and attenuation law of stress wave in jointed rock mass are the problems that need to be paid attention to in the research of rock dynamics and engineering application^1^.

During the blasting excavation of engineering rock mass, the excavation is not completed at one time, but the blasting operation is carried out step by step. Multiple blasting loads will inevitably lead to the damage of filled joints in the trenchless zone, resulting in the reduction of their strength^2^. Accordingly, the damage and deterioration of filled joints will reduce the overall strength and stability of rock mass, and will aggravate the attenuation of stress wave in rock mass. Therefore, it is necessary to study the dynamic characteristics of filled joints under dynamic load disturbance and its influence on stress wave propagation.

At present, the experimental research on the mechanical properties of joints is mainly through uniaxial compression^3–5^, shear^6–8^ and dynamic impact^9–14^. More researches have been done on unfilled joints and fractured joints, but there are relatively few experimental studies on filled rock joints. The impact test of artificial jointed rock specimens is mostly carried out by the split Hopkinson pressure bar (SHPB) device, which is used to explore the influence of filling materials, thickness of filled joints, various angles of filled joints, water content of fillings, loading rate and other characteristics on the dynamic properties and stress wave transmission of rock^2,15–18^. However, there is a lack of research on dynamic characteristics of filled joints under cyclic dynamic load. As for cyclic dynamic loading, researchers mainly studied intact rocks, analyzed the influence of cyclic impact loading on the dynamic characteristics of rocks, and established the characterization method of cumulative damage of rocks under cyclic impact^19–21^. These studies on rock cumulative damage under cyclic impact can provide reference for the research on dynamic characteristics of filled joints under cyclic dynamic load.

Moreover, some research has been carried out on the compressive mechanical properties of filled joints, but most of them focus on static or dynamic unilateral experimental analysis. Few of these studies conducted comparative analysis on filled joints under static and dynamic compression at the same time. Beside, most of them only carried out experimental studies under single factor, and relatively few of them considered filling materials and filling thickness. Therefore, in consideration of the two factors of filling material characteristics and thickness of filled joint layer, which have great influence on mechanical properties of joints, Chai et al.^2^ used universal testing machine and SHPB device to conduct experimental research on compressive mechanical properties of filled joints.

On the basis of the above research, this paper makes a simulation test analysis on the cumulative damage caused by multiple explosion shocks to the filled joints of rock mass in the elastic zone. Through dynamic and static compression tests, the strength characteristics, deformation characteristics, fluctuation characteristics and energy dissipation of filling joints under static compression and dynamic impact compression are studied and analyzed respectively.

## Multiple impacts of filled jointed rock specimens

### Filled jointed rock specimens and self-made falling weight impact device

**Figure 1:**
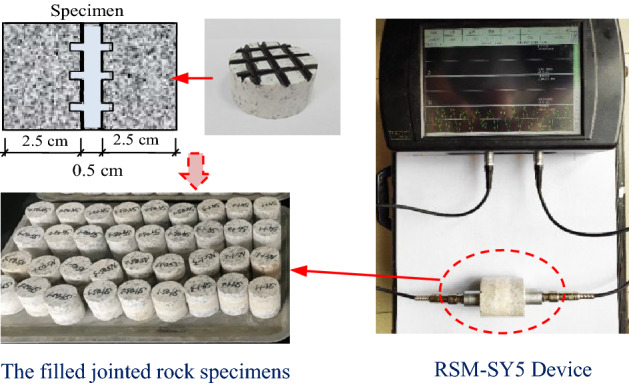
Filled rock joint specimen and wave velocity testing.

This present study is carried out on the basis of previous research results, and the specimen making method is consistent with the specimen in article^2^. In the previous study^2^, the joint layer was filled with cement mortar, gypsum mortar, lime mortar and mud-sand mixture, with filling thickness of 2, 5 and 10 mm respectively. The results show that the strength of cement mortar filling layer is too high and the joint properties are not obvious. The strength of the mud-sand mixture is too low and will be destroyed at the initial stage of compression. The brittleness of gypsum mortar is too strong, and the filling layer will be destroyed and scattered under the first pre-impact. Therefore, in order to better realize the research purpose of this paper, the influence of different filling materials is no longer considered in the experiments, and lime mortar is taken as the filling material. Moreover, the joint filling layer in practical engineering is mostly composed of weathered rock particles and clay, which is consistent with the properties of lime mortar. In addition, the filling thickness is also one of the main factors affecting the mechanical properties of jointed rock samples^2^. In order to make a better single factor analysis, only one thickness filled joint is tested in this paper. In the existing literatures^1,2,17,18^, the ratio between the thickness of joint filling layer and the total thickness of sample is mostly 1/15–1/4. In this paper, 5 mm is selected as the filling thickness, and its ratio to the total thickness of the sample is 1/7, which falls within the above range. As shown in Fig. 1, the granite on both sides is processed into cylindrical specimens with a diameter of 50 mm and a height of 25 mm. One end face of the rock block is regularly polished and grooved to make the surface roughness basically consistent. Every two granite blocks are mixed with filling materials to form a jointed rock specimen. In order to ensure the consistency of the specimen and improve the reliability of the test, the wave velocity of the rock specimen is measured by RSM-SY5 sonic instrument after the specimen is made, and the specimens with large deviation are removed.

**Figure 2:**
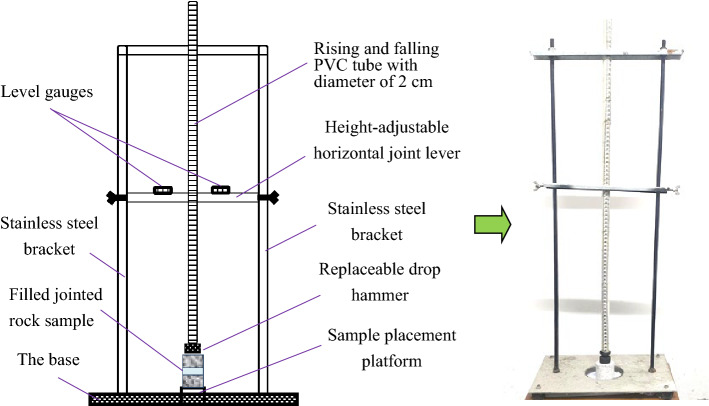
Self-made falling weight impact device.

After the specimens are selected, a self-made simple drop hammer impact device shown in Fig. 2 is used to conduct pre-impact test on the filled jointed rock specimens. During pre-impact loading, the impact height is set as 60 cm, and the weight of the drop hammer is 150 g. The pre-impact on the filled rock specimens is realized through the free fall of the drop hammer. In the process of pre-impact loading, the rock specimen will be damaged to a certain extent but not destroyed. The cumulative pre-impact tests on the filled rock joints specimens are carried out with this device with different impact times (1, 2, 3, 4 and 5 times). Meanwhile, RSM-SY5 acoustic wave tester is used to measure the wave velocity of the specimens before and after each pre-impact. The variation of wave velocity can reflect the damage change rule of filled joints under cumulative pre-impact to a certain extent. In the comparative test, the impact times of the undisturbed samples without pre-impact are marked as 0 times. Multiple tests show that when the rock specimen is impacted for 6 times, the filled layer of rock specimens is totally destroyed. Thus, only the cumulative impact results of 0–5 times are analyzed in this present study**.**

### Damage law of rock specimens after multiple impacts

In the process of cumulative pre-impacts, micro-cracks in the filled joints continue to develop and expand, and damage intensifies, resulting in the deterioration of joint integrity, the change of elastic modulus, wave velocity and other parameters of rock samples. Because the attenuation of ultrasonic velocity is the macroscopic expression of damage degree in solid, it is a concise and effective method to describe the damage variables of filled jointed rock specimens under cumulative pre-impact by measuring the attenuation of wave velocity. Therefore, in order to study the damage evolution characteristics of filled joints in the process of cumulative impact, ignoring the small change of density before and after impact damage of the rock specimens, the damage degree *D*_*n*_ of filled joint rock samples can be defined. According to the equivalent strain hypothesis, assuming that the average elastic modulus of the filled jointed rock specimen without damage is $\overline{E}$ and the average elastic modulus after *n* times of pre-impact damage is $\overline{{E_{n} }}$, the damage degree *D*_*n*_ of the filled joints after the *n*th impacts:

1 $$D_{n} = 1 - \frac{{\overline{{E_{n} }} }}{{\overline{E} }}$$

It is assumed that the wave velocity of the rock specimen without damage is *v*_0_ and the wave velocity after the *n*th impact damage is *v*_*n*_. According to the relation between the elastic modulus of solid material and density and wave velocity, $E = \rho v^{2}$, the damage variable *D*_*n*_ can be expressed as:

2 $$D_{n} = 1 - \frac{{v_{n}^{2} }}{{v_{0}^{2} }}$$

The acoustic wave velocity method has been widely used in geotechnical engineering to measure rock damage. This method is simple and easy to operate, with low degree of external interference. Therefore, according to formula (2), the change of ultrasonic wave velocity can directly and effectively reflect the damage evolution law of filled joints after cumulative pre-impact.

The wave velocity values of filled jointed rock specimens under different cumulative impacts are tested. Three specimens are taken for the each condition, and wave velocity of each specimen is measured for three times. The results with large variance are excluded, and the average value of wave velocity and corresponding cumulative damage value under each working condition are calculated, as shown in Table 1. At the same time, in the process of impact disturbance, the same pre-impact test and wave velocity test are carried out on the homogeneous granite specimen. The granite specimen has the same size as the filled jointed rock specimens and is consistent with the rock material on both sides of the joint. It can be seen from Table 1 that the damage of filled jointed rock specimens under cumulative impact mainly occurs in the filled layer.

**Table 1:** Physical and mechanical parameters of rock and filled joint materials.

The specimens	Density (kg/m^3^)	The average value of wave velocity $\overline{v}_{n}$ (m/s) and cumulative damage degree $D_{n}$										
		$\overline{v}_{0}$	$\overline{v}_{1}$	$D_{1}$	$\overline{v}_{2}$	$D_{2}$	$\overline{v}_{3}$	$D_{3}$	$\overline{v}_{4}$	$D_{4}$	$\overline{v}_{5}$	$D_{5}$
Filled jointed rock sample	2298	1957	1452	0.450	1250	0.592	1184	0.634	1071	0.700	1009	0.734
Lime mortar sample	1593	370	256	0.521	228	0.620	196	0.719	178	0.769	174	0.779
Granite sample	2430	3368	3347	0.012	3318	0.029	3282	0.050	3230	0.080	3142	0.130

## The static compression results and analysis

**Figure 3:**
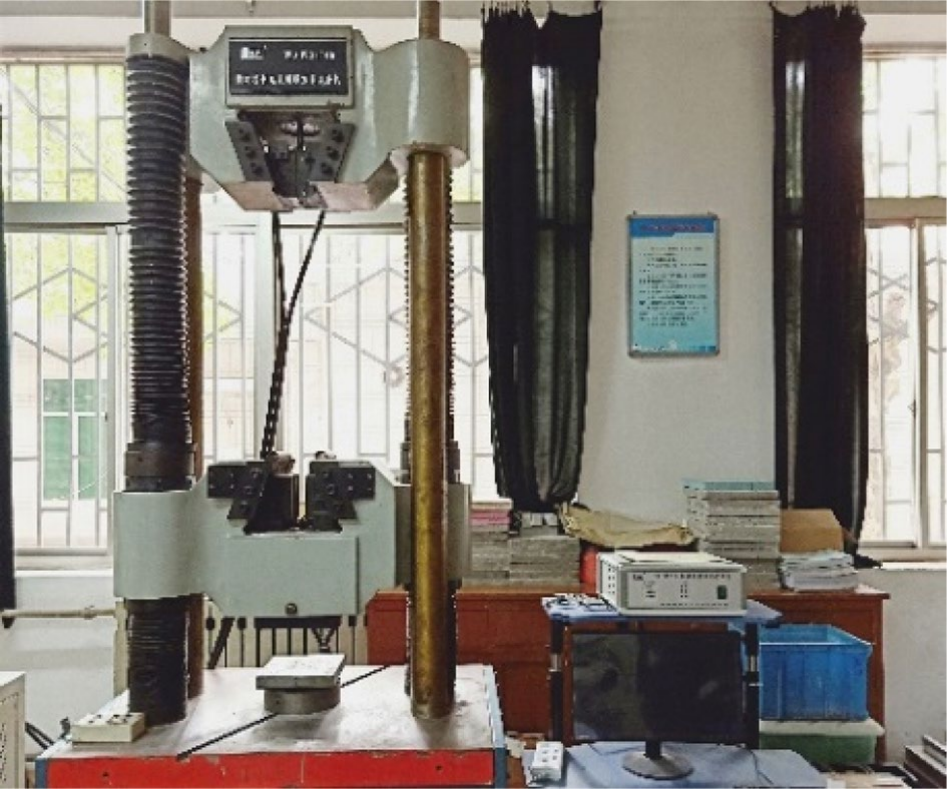
The WAW31000 microcomputer control universal testing machine.

The static compression test is carried out by WAW31000 microcomputer controlled electro-hydraulic servo universal testing machine, as shown in Fig. 3. The test force loading control is adopted during the test, and the loading rate was 50 N/s.

### Analysis of static compressive strength and deformation characteristics

**Figure 4:**
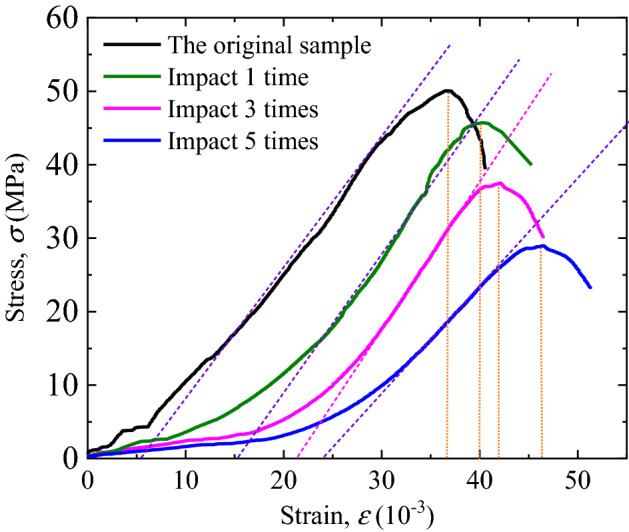
The Static compressive stress–strain curves of filled joints.

The stress–strain curves of filled jointed rock specimens under four different pre-impact times are obtained through static compression tests, as shown in Fig. 4.

The stress value at the point where it reaches the maximum value of the curve is defined as the peak stress, and the strain corresponding to the peak stress is the peak strain *ε*_*p*_. As the number of pre-impact increases, the peak stress of the specimen under uniaxial compression decreases while the corresponding strain increases. After the first impact, the peak stress of the specimen decreases significantly, about 9% of the strength of the undisturbed specimen, and the corresponding strain increases by about 9%. Similarly, after three times of impact, the peak stress of rock specimen is 37 MPa, which is 26% less than that of undisturbed rock specimen. Nevertheless, the peak strain only increases by about 5% compared with that after the first impact. After five times impact, the peak stress of rock specimen is 29 MPa, which is 43% less than that of undisturbed rock specimen, and the peak strain increases by about 10% compared with the peak strain after three times impact.

### Influence of multiple impacts on deformation characteristics of filled joints

According to the previous research results^2^, the whole compression process of filled joints under uniaxial compression can be roughly divided into three stages: joint compression stage, joint failure stage and rock compression stage. From the stress–strain curves shown in Fig. 4, it can be seen that the curves corresponding to different pre-impact times have significantly different variation rules in the stress growth stage (rising section of the curve). In the absence of impact disturbance, the curve first appears a small slow growth stage (joint compression section) accompanied by slight oscillation (joint failure section). Then it enters a stage of linear growth (elastic compression section).

With the increase of the number of pre-impact, the slow growth stage of the curve gradually lengthened, which means that the increase of stress is small but the increase of strain is large. In other words, the cumulative impacts have a significant influence on the mechanical properties of the joint filling layer, which will cause the structure of the joint layer to be damaged. Therefore, in the joint compression stage, it shows a weak increase in strength and a significant increase in strain.

As shown in the figure, each curve has an obvious linear growth section, and the slope of this linear section reflects the elastic modulus of rocks on both sides of the joint. Under the impact of 0, 1 and 3 times, the slopes of the linear segments of the three curves are basically close, indicating that the damage of the rocks on both sides of the joint under these three working conditions is small and can be ignored. The disturbance caused by impact mainly acts on the joint layer. However, in the stress–strain curve after 5 times impact, the slope of the linear section is significantly lower than that of the other three curves, indicating that in addition to the obvious damage of the joint layer, there is also a certain cumulative damage to the rocks on both sides of the joint. This rule can also be verified by the wave velocities measured in Table 1 under different impact times. In Table 1, the wave velocity of the lime mortar specimen of filling layer decreases rapidly under the first impact and then decreases slowly. The reduction of wave velocity of rock specimens on both sides of the joint is small in the first to fourth times of impact, but the wave velocity decreases obviously after the fifth time of impact. At this time, the cumulative damage of rock is obviously reflected in the change of wave velocity.

If the coordinate where the extension line of linear section of each curve intersects the horizontal axis is defined as damage strain *ε*_*d*_, the value of damage strain will reflect the unrecoverable deformation of filled rock joints specimens before reaching peak strain *ε*_*p*_. In this way, the difference between peak strain and damage strain can be defined as the elastic strain of the filled rock joints specimens, *ε*_*e*_, which is:

3 $$\varepsilon_{e} = \varepsilon_{p} - \varepsilon_{d}$$

The strain values obtained from the test are shown in Table 2.

**Table 2:** Static compression strain statistics of rock specimens after different cumulative impact.

	0	1	2	3	4	5
Peak strain, *ε*_*p*_	36.64	40.11	42.05	46.42	36.64	40.11
Damage strain, *ε*_*d*_	4.88	14.95	21.26	23.82	4.88	14.95
Elastic strain, *ε*_*e*_	21.76	25.16	20.79	22.60	21.76	25.16

It is obvious from Table 2 that cumulative impact has little influence on the elastic strain of the filled rock joints specimens, which remains between 20 and 25. On the contrary, the filled layer has a very obvious response to cumulative impact. The damage strain increases by 200% under one impact, and then gradually increases. In the case of five impacts, it reaches 5 times of the damage strain of the rock specimens without pre-impact. Due to the different responses of the filled layer and the rock on both sides to the cumulative impact, the filled rock joints specimens formed by the combination of the two have more complex mechanical properties. As the conclusion obtained in the previous study^2^, the thickness of filled joints and the mechanical properties of the filling layer have a great influence on the mechanical properties of the filled rock joints specimens. The cumulative damage effect under the comprehensive consideration of all factors is worth further study.

## Dynamic compression results of rock specimens after impacts

**Figure 5:**
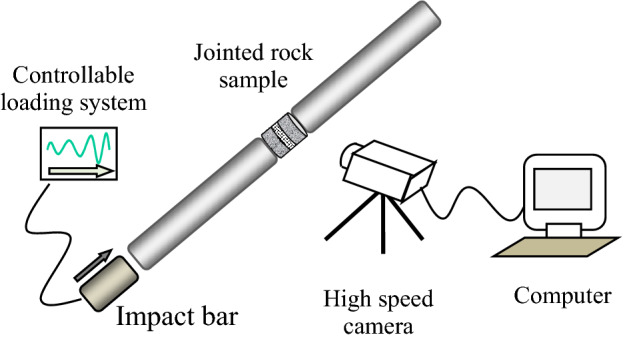
Schematic diagram of SHPB test device with a high speed camera.

The split Hopkinson pressure bar (SHPB) device is used to conduct dynamic impact test on the specimens after multiple pre-impacts, and the dynamic characteristics of cumulative damage of filled jointed rock specimens are studied. As shown in Fig. 5, the device is composed of an impact bar, an incident bar, a transmission bar and an absorption bar. Each bar is made of 50 mm diameter steel with a density of 7800 kg/m^3^, an elastic modulus of 210 GPa and a wave speed of 5227 m/s. The lengths of impact bar, incident bar, transmission bar and absorption bar are 0.4 m, 3.5 m, 2.5 m, and 1.5 m respectively. The symmetrical patch is adopted to eliminate the error caused by bending deformation of elastic bar during impact process. Besides, in order to prevent the superposition of the waveforms of incident and reflected waves, the strain gauge is pasted on the incident bar 2300 mm away from the contact surface of specimen and the transmission bar 600 mm away.

**Figure 6:**
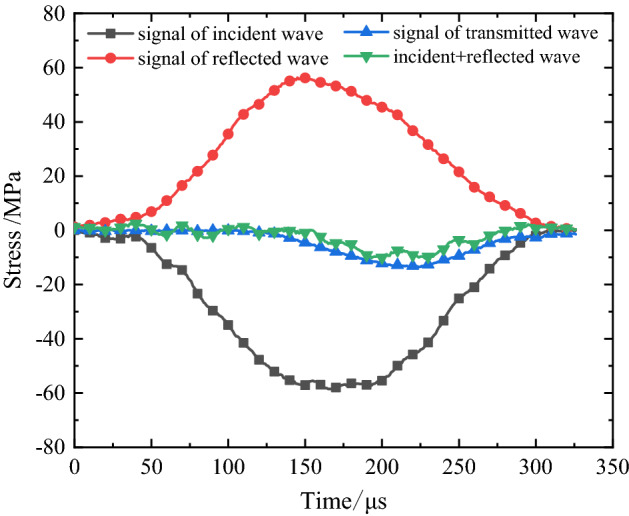
Stress balance diagram of jointed rock sample under SHPB impact load.

The filled jointed rock specimen is a kind of brittle material with small failure strain. Therefore, in the test, rubber shaping plate is used as pulse shaper to make the incident wave smooth, so as to meet the stress uniformity and eliminate the dispersion effect of stress wave propagation. After translating the jump time of incident, reflected and transmitted signals, the schematic diagram of stress balance at both ends of the sample can be obtained. Figure 6 shows the relationship between stress and time of the sample without pre-impact under impact load. It can be seen from the figure that the superposition of incident stress and reflection stress is close to the transmission stress, which meets the dynamic equilibrium condition, indicating that the test data obtained under SHPB impact load are valid.

During the impact process, a Photron FASTCAM Mini UX high-speed camera is used to record the impact process of the sample. The impact air pressure in the test is set at 0.2 MPa, and the measured average velocity of the incident bar is 3.4 m/s. The test is repeated three times for each group of specimens to ensure that the amplitude of each incident wave is approximately equal. After sorting out the test results, the dynamic mechanical properties of filled joints under cumulative impact are analyzed from the aspects of joint strength, wave characteristics, energy transfer and impact failure process.

### Strength and deformation of filled joints under impact compression

During the dynamic impact test, the strain data, including incident strain *ε*_*i*_, reflected strain *ε*_*r*_and transmitted strain *ε*_*t*_, at any time can be recorded by a collection device connected to the strain gauges. According to the three-wave method^22^, the mean strain *ε*(*t*), stress *σ*(*t*) and strain rate $\dot{\varepsilon }(t)$ of jointed rock samples can be calculated:

4 $$\varepsilon (t) = \frac{C}{L}\int_{0}^{t} {(\varepsilon_{i} - \varepsilon_{r} - \varepsilon_{t} )} d_{t}$$

5 $$\sigma (t) = \frac{{AE_{b} }}{2S}(\varepsilon_{i} + \varepsilon_{r} + \varepsilon_{t} )$$

6 $$\dot{\varepsilon }_{(t)} = \frac{c}{l}(\varepsilon_{i} - \varepsilon_{r} - \varepsilon_{t} )$$

where *A* is the cross-sectional area of the pressure bar, *S* is the cross-sectional area of the sample, *E*_*b*_ is the elastic modulus of the pressure bar, and *C* is the wave velocity in the pressure bar. Equations (4) and (5) are combined to eliminate time *t*, and the dynamic stress–strain relationship of rock samples filled with joint under impact compression can be obtained.

**Figure 7:**
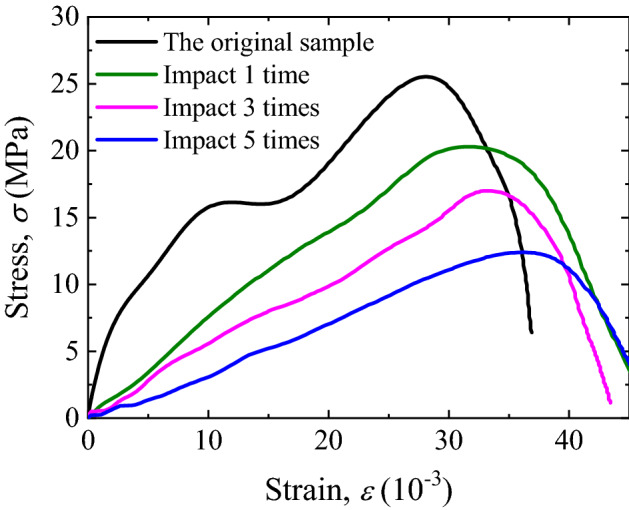
Dynamic stress–strain curves of filled jointed rock specimens after accumulative pre-impacts.

The dynamic stress–strain curve of the filled joints under impact compression is obtained accordingly, as shown in Fig. 7.

It can be seen from Fig. 6 that the dynamic peak stress of the specimen decreases with the increase of the number of accumulative pre-impacts. Besides, the strength of the specimen decreases by basically the same amplitude after the 1, 3 and 5 times of pre-impacts. This is because the strength of the lime mortar filling layer is lower than that of the rocks on both sides, and the internal structure has been destroyed at the first pre-impact. After that, with the increase of impact times, the damage accumulates and the strength gradually decreases.

The comparison between Figs. 4 and 7 shows that the dynamic compression characteristics of filled joints are significantly different from the static compression. The initial section of the static compression stress–strain curve has a slow growth stage, which reflects the compression deformation process of the joint layer. However, the initial section of the dynamic compression stress–strain curve of each specimen is approximately a straight line, indicating that the filled rock joints specimens is in a state of linear elastic compression, with good linear deformation characteristics. The main reason is that the the deformation process of filled jointed rock samples needs to adapt to the high strain rate of 10^3^ s^−1^ under SHPB dynamic impact. When the impact stress wave with high strain rate acts on the filled jointed rock specimens, the compression deformation process of the joint layer is too fast, and the stress in the joint is always in balance with the rock specimen on both sides, so the joint layer and the rock on both sides show good integrity together.

In addition, the slope of the straight line segment of each curve reflects the elastic modulus of the whole rock specimen. When the stress rises to a certain value, the curve drops sharply, indicating that a part of the shear failure occurs along the filled layer under impact compression.

### Influence of damage degree of filled jointed rock specimens on compression characteristics

**Figure 8:**
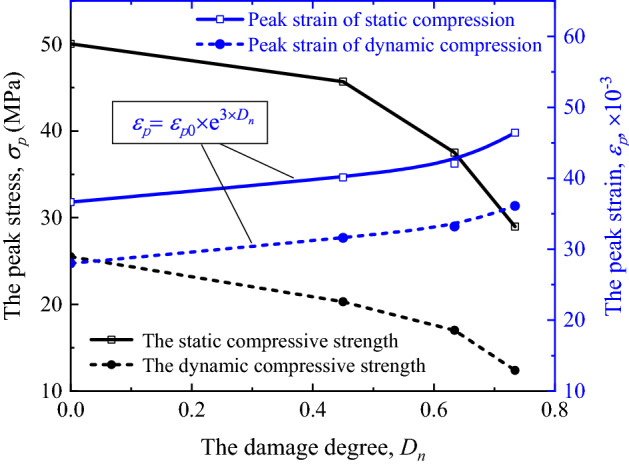
Influence law of damage degree of filled rock joints specimens on compression characteristics.

In order to better analyze the influence of cumulative impact on the compressive mechanical properties of filled jointed rock specimens, the peak strength and peak strain of static compression and dynamic compression under each working condition are sorted with the damage degree mentioned in “Multiple impacts of filled jointed rock specimens” section as the independent variable, as shown in Fig. 8.

It can be seen from the figure that the variation law of peak strain under static compression with cumulative damage degree is basically consistent with that under dynamic compression, and the two curves are nearly parallel. By fitting the curve of peak strain variation with damage degree, the following formula can be obtained:

7 $$\varepsilon_{p} = \varepsilon_{p0} \cdot e^{{3 \cdot D_{n} }}$$

where $\varepsilon_{p0}$ is the corresponding value when *D*_*n*_ = 0, that is, the peak strain of undamaged filled rock joints specimens. The fitting degree *R*^2^ of the formula for the dynamic compression strain curve is 0.8764, and the fitting degree of the dynamic curve is about 0.9126, indicating that the fitting result of the curve is good. The curves show that the compressive strain of filled jointed rock specimens after cumulative impacts increases exponentially with damage degree.

Nevertheless, the influence law of cumulative damage degree on peak strength is slightly different, and the first half of the two curves is nearly parallel. That is, when the damage degree is relatively small (< 0.5), the variation law of static and dynamic strength with damage degree is similar, and the static compressive strength value is about twice that of the dynamic strength. When the damage degree is relatively large, the static strength decays faster than the dynamic strength, but its value is still much higher than that of the dynamic strength, even higher than that of the dynamic strength of undamaged rock specimen.

### Wave characteristics of filled joints under impact compression

**Figure 9:**
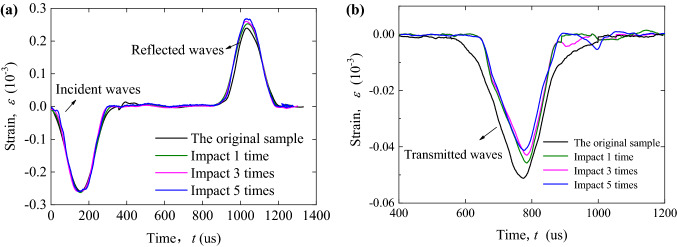
The waveform curves of the jointed rock specimens filled with clay-sand mixture under one-dimensional impact. (**a**) The incident wave and reflected wave; (**b**) The transmitted wave.

Figure 9 shows the waveform curves of incident wave, reflected wave and transmitted wave in rock specimens with cumulative impact times of 1, 3 and 5 respectively.

It is obvious from the figure that under different cumulative pre-impact times, the waveforms of transmitted and reflected waves change little, but the amplitude changes obviously. Specifically, with the increase of the number of pre-impacts, the amplitude of reflected wave increases gradually, while that of transmitted wave decreases gradually. The above rule can be explained by the change of wave impedance. In the previous research paper^2^, the reflection coefficient of stress wave of filled jointed rock specimens in SHPB test was given as follows:

8 $$R = \frac{{z_{p1} - z_{p2} }}{{z_{p1} + z_{p2} }}$$

where *z*_*p*1_ and *z*_*p*2_ are the wave impedance of the compression bar and rock specimen respectively. Obviously, the wave impedance of the bar, *z*_*p*1_, remains unchanged. The average wave impedance of a rock specimen consisting of granite blocks and filled layers is the product of the average density and average wave velocity of the rock specimen, i.e., $\overline{{z}_{p2}}=\overline{\rho }\cdot \overline{v}$. As seen from Table 1, when the number of pre-impact increases, the cumulative damage of rock specimen increases while the average wave velocity decreases significantly, resulting in a decrease in the average wave impedance. According to Formula (8), when the average wave impedance of the rock specimen decreases, the reflection coefficient increases.

### Energy dissipation of filled joints under impact compression

According to the one-dimensional stress wave theory and law of conservation of energy, the incident energy, *E*_*i*_, the reflected energy, *E*_*r*_ and the transmitted energy, *E*_*t*_*,* can be calculated, as follow:

9 $$E_{m} = S \cdot E \cdot C_{0} \int_{0}^{t} {\varepsilon_{m}^{2} } d_{t}$$

where *ε*_*m*_ denotes the strain of each wave; *m* is *I*, *r* or *t*, represents incident wave, reflected wave and transmitted wave, respectively; *S* denotes the section area of the pressure bar; *E* denotes the elastic modulus of the bar, and *C*_*0*_ is the velocity of stress wave across the pressure bar. According to the law of energy conservation, the energy dissipated on the jointed rock sample, *E*_*s*_, can be calculated as:

10 $$E_{s} = E_{i} - E_{r} - E_{t}$$

**Figure 10:**
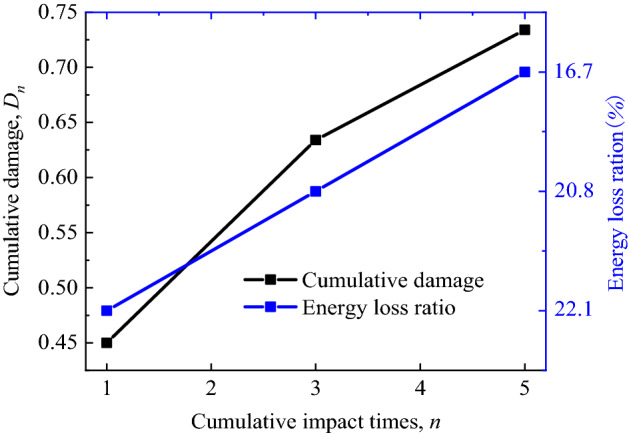
Cumulative damage and energy consumption ratio.

The influence of impact compression on stress wave energy transmission in the filled joints can be analyzed by the defined energy consumption ratio $E_{{\text{s}}} /E_{i}$. Accordingly, the distributions of cumulative damage and energy consumption ratio under different pre-impact times are obtained, as shown in Fig. 10.

It is obvious from the figure that there is a negative correlation between energy consumption ratio and cumulative damage degree. From the perspective of microstructure, with the increase of cumulative pre-impact times, the damage degree inside the filled joints is bound to increase, that is, the micro-cracks gradually continues to expand and connect, so as to reduce the energy consumption required for the failure of the filled jointed rock sample under impact load. Ignoring the energy loss of stress wave propagating in the compression bar, the vast majority of *E*_*s*_ is mainly used for the damage and failure of filled jointed rock specimens. Therefore, the value of $E_{{\text{s}}} /E_{i}$ reflects the proportion of energy required for joint failure.

### Dynamic failure characteristics of rock specimens under impact compression

**Figure 11:**
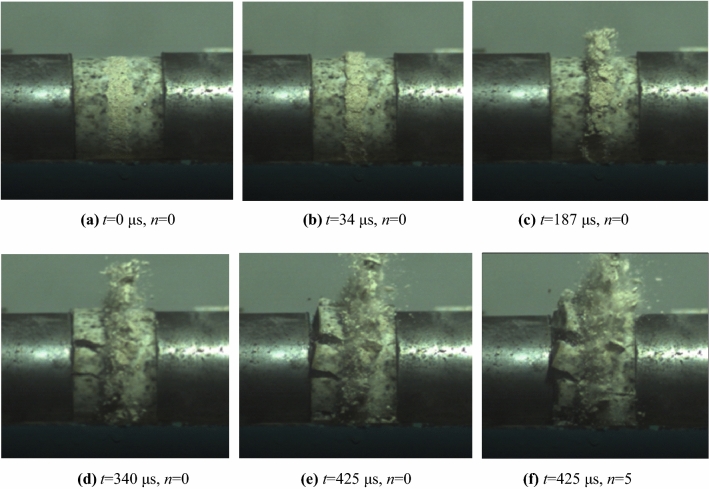
Photos of impact failure process of filled jointed rock specimens.

In SHPB impact test, the crack development process and failure state of filled rock joints specimens during impact were recorded by a high-speed camera. Figure 11a–e shows the failure process of filled jointed rock specimen without pre-impacts (i.e., *n* = 0) during impact. Where, Fig. 11a is the initial state of the sample, and the time when the stress wave is about to arrive at the sample is set as t = 0. According to this, the sample photos at other times can be obtained by shifting time axis. Combined with the stress–strain curve shown in Fig. 7, the failure process of filled jointed rock specimens can be divided into the stage of overall elastic deformation (Fig. 11b), the stage of failure of joint filling layer (Fig. 11c), the stage of fracture propagation (Fig. 11c) and the stage of overall failure stage (Fig. 11d). Due to the large number of micropores and relatively loose structure of mortar filling the joint layer, the pores inside the joint layer are closed firstly under impact load, and the joint layer is compressed to a certain extent, resulting in slight bulge on the side. Then the rocks on both sides have a certain elastic deformation, which lasts for a short time. As shown in Fig. 11a,b, the filling layer in Fig. b has a certain amount of compression and slight bulge compared with the initial state shown in Fig. a. It can be seen that the deformation in the initial compression stage mainly occurs in the filling layer. With the continuous impact, the external surface of the filled joints is uplifted and accompanied by debris shedding, and then the filling joint is gradually destroyed. In the late stage of filling joint failure, microcracks begin to appear in the rocks on both sides, and the microcracks continue to expand and connect, resulting in the failure of the rocks on both sides.

In order to compare the final failure modes of filled rock joints specimens subjected to dynamic impact with different impact times, the final failure state diagrams of filled jointed rock specimens under 0 and 5 times cumulative impacts are analyzed, as shown in Fig. 11e,f. Obviously, the number of cracks on the surface of the sample after cumulative impact increases significantly, and the crack direction forms a certain inclination angle with the axis direction of the filled joint rock. In addition, the degree of fragmentation of filled jointed rock specimens is intensified, and the degree of crushing splash of joint filling layer is intensified. All of these are due to the cumulative impact causes structural damage to the joint filling layer and weakens the adhesion force between particles, thus reducing the impact resistance of the filled jointed rock (Supplementary Information)^23^.

## Conclusions

In this present study, the compression tests are carried out on filled jointed rock specimens with different cumulative pre-impacts by universal testing machine and SHPB device, and the effects of cumulative impact on static and dynamic compression characteristics of filled joints are analyzed accordingly. The main conclusions are as follows:

1. Cumulative impact will cause obvious damage to the filled jointed rock specimens, and the damage mainly appears in the filled layer. The damage strain can be clearly seen in the stress–strain curve, which means the weak increase of strength and the significant increase of strain at the joint compression stage.
2. The static and dynamic compressive strength of the filled joints decreases with the increase of cumulative damage degree. On the contrary, the peak strain of static and dynamic compression increases with the increase of cumulative damage degree, and the growth law satisfies the exponential function,$\varepsilon_{p} = \varepsilon_{p0} \cdot e^{{3 \cdot D_{n} }}$.
3. In the case of the same incident wave amplitude, with the increase of cumulative damage degree, the reflected wave amplitude increases slightly while the transmitted wave amplitude decreases gradually. The stress wave propagation characteristics are closely related to the reduction of impedance of jointed rock specimens.
4. Under impact compression, the energy dissipation ratio $E_{{\text{s}}} /E_{i}$ decreases with the increase of cumulative damage degree of filled joints, which confirms the engineering fact that filled joints are more prone to damage after cumulative impact.

## Supplementary Information

Supplementary Information.

## Data Availability

The datasets used and/or analysed during the current study available from the corresponding author on reasonable request.
